# Structured sedation programs in the emergency department, hospital and other acute settings: protocol for systematic review of effects and events

**DOI:** 10.1186/2046-4053-2-89

**Published:** 2013-10-01

**Authors:** Siobhán McCoy, Abel Wakai, Carol Blackburn, Michael Barrett, Adrian Murphy, Maria Brenner, Philip Larkin, Gloria Crispino-O’Connell, Savithiri Ratnapalan, Ronan O’Sullivan

**Affiliations:** 1 Department of Emergency Medicine, Our Lady’s Children’s Hospital, Crumlin, Dublin 12, Ireland; 2 Paediatric Emergency Research Unit (PERU), National Children’s Research Centre, Our Lady’s Children’s Hospital, Crumlin, Dublin 12, Ireland; 3 Emergency Care Research Unit (ECRU), Division of Population Health Sciences (PHS), Royal College of Surgeons in Ireland (RCSI), 123 St Stephen’s Green, Dublin 2, Ireland; 4 School of Nursing, Midwifery and Health Systems, University College Dublin, Belfield, Dublin 4, Ireland; 5 Divisions of Paediatric Emergency Medicine, Clinical Pharmacology and Toxicology, Department of Paediatrics and Dalla Lana School of Public Health, The Hospital for Sick Children, University of Toronto, 555 University Avenue, Toronto, ON M5G 1X8, Canada; 6 Department of Paediatrics, School of Medicine & Medical Science, University College Dublin, Belfield, Dublin 4, Ireland; 7 Department of Emergency Medicine, Cork University Hospital, Wilton, Cork, Ireland

**Keywords:** Procedural sedation, Conscious sedation, Sedation program, Pediatric, Sedation education

## Abstract

**Background:**

The use of procedural sedation outside the operating theatre has increased in hospital settings and has gained popularity among non-anesthesiologists. Sedative agents used for procedural pain, although effective, also pose significant risks to the patient if used incorrectly. There is currently no universally accepted program of education for practitioners using or introducing procedural sedation into their practice. There is emerging literature identifying structured procedural sedation programs (PSPs) as a method of ensuring a standardized level of competency among staff and reducing risks to the patient. We hypothesize that programs of education for healthcare professionals using procedural sedation outside the operating theatre are beneficial in improving patient care, safety, practitioner competence and reducing adverse event rates.

**Methods/Design:**

Electronic databases will be systematically searched for studies (randomized and non-randomized) examining the effectiveness of structured PSPs from 1966 to present. Database searches will be supplemented by contact with experts, reference and citation checking, and a grey literature search. No language restriction will be imposed. Screening of titles and abstracts, and data extraction will be performed by two independent reviewers. All disagreements will be resolved by discussion with an independent third party. Data analysis will be completed adhering to procedures outlined in the *Cochrane Handbook of Systematic Reviews of Interventions*. If the data allows, a meta-analysis will be performed.

**Discussion:**

This review will cohere evidence on the effectiveness of structured PSPs on sedation events and patient outcomes within the hospital and other acute care settings. In addition, it will examine key components identified within a PSP associated with patient safety and improved patient outcomes.

**Trial registration:**

PROSPERO registration number: CRD42013003851

## Background

### Description of the condition

Pain management is inconsistent, inadequate or both, throughout the hospital setting [1-3]. Patient surveys and complaints reveal inadequate pain management as a common issue in many healthcare systems [4,5]. Meanwhile, approximately 70% of patients presenting to emergency departments (EDs) have a chief complaint related to pain [6-8]. Additionally, in the ED setting many patients require relatively painful diagnostic and therapeutic procedures, such as venepuncture, venous cannulation, wound closure, and closed manual reduction of fractures and dislocated joints. These procedures are painful, distressing and are associated with significant anxiety, particularly in children. Procedural pain is an aspect of pain management that healthcare professionals can control in the ED, and the American Academy of Pediatrics (AAP) states that pain secondary to medical or nursing procedures can be greatly reduced or avoided [9].

#### Description of the intervention

Procedural sedation is the use of sedative, analgesic and dissociative agents, which facilitate the performance of interventions by causing a state of sedation, alleviating pain while enabling the patient to maintain airway reflexes and respiratory drive [10,11]. The appropriate administration of sedative and analgesic agents enhances the comfort and acceptance of diagnostic and therapeutic procedures [12]. However, many of these agents retain the capacity to produce serious and potentially life-threatening adverse effects [13].

Over the last two decades procedural sedation has become a common treatment option in both the adult and pediatric ED setting, facilitating the performance of procedures, such as laceration repair and fracture reduction, that otherwise would need to be performed under general anesthesia in the operating room setting [14-17]. Due to the increased demand for, and the requirements of, a procedural sedation service outside the operating theatre, it is no longer feasible or appropriate for a high quality service to be provided and maintained by anesthetists alone [18]. Procedural sedation guidelines provided by various governing bodies are, by their nature, non-binding and are not intended to standardize practice [19]. This lack of uniformity and standardization has led to varying practices, which are a potential risk to patient safety.

Structured programs of education and guidelines are widely advocated as a method of reducing clinical risk and improving quality of care provided to patients; however, there are few studies investigating outcomes associated with the use of guidelines and education [20]. Borland *et al*. [15] reported that the development of an evidence-based joint clinical practice guideline (CPG) with education and credentialing packages for the Paediatric Research in Emergency Departments International Collaborative (PREDICT) network in Australia would standardize practice and may reduce patient risk. Coté *et al*. [21,22] identified an association between the adherence to formal guidelines and the minimization of clinical risk. There remains a dearth of evidence examining the effects of differing training methods for procedural sedation on the quality of the sedation event [20,23]. In a formal evaluation of a hospital-wide structured model of procedural sedation, Hoffman *et al*. [13] reported an association between adherence to the program and a reduction in procedural sedation-related complications.

#### Why is it important to do this review?

Most guidelines of sedation by non-anesthetists lack clarity and remain inconsistent on the subject of staff preparedness or training. There are currently no indicators or measures of quality available for sedation services provided outside the operating theatre. This results in poor quality sedation provision embodied by varying levels of competence, reported adverse events and sedation practices within specialties such as emergency medicine, dentistry, oncology and radiology [19,24-26]. There is no universally accepted program for education and accreditation for procedural sedation within the ED setting. Definitive conclusions regarding the benefit of structured procedural sedation programs (PSPs) to patient care, safety, practitioner competence and lower adverse event rates are hampered by gaps in the literature [27].

### Objectives

We hypothesize that programs of education for healthcare professionals using procedural sedation outside the operating theatre are beneficial in improving patient care, safety, practitioner competence and reducing adverse event rates.

## Methods/Design

The review team is multidisciplinary, and includes content experts, a reference librarian, clinical researchers and systematic review experts. The review is registered with the PROSPERO international prospective register of systematic reviews (registration number: CRD42013003851). We will conduct a systematic review adhering to the criteria outlined below.

### Criteria for considering studies for this review

#### Types of studies

• Randomized controlled trials (RCTs)

• Cluster randomized controlled trials (C-RCTs)

• Non-randomized control trials (NRCTs)

• Non-randomized cluster controlled trials

• Controlled before and after (CBA) studies – prospective observational reviews

• Interrupted time series (ITS) studies.

#### Types of clinical setting

• EDs

• Other hospital inpatient settings, for example diagnostic imaging departments, endoscopy suites and coronary care units

• Sedation units

• Other acute care settings, for example urgent care centers

• Dental surgeries/clinics.

#### Types of participants

• Patients who undergo procedural sedation in the ED and/or other hospital settings and acute care facilities

• Both children and adults will be included.

### Types of intervention

The use of PSPs for all non-anesthetist healthcare practitioners administering procedural sedation to patients.

### Types of comparison

Provision of sedation by non-anesthetists without the use of a PSP for healthcare staff.

### Types of outcome measures

#### Primary outcomes

• Respiratory/airway compromise requiring ventilatory support

• Patient satisfaction rates as defined by the study authors

• Practitioner competence.

#### Secondary outcomes (as defined by study authors)

• Evidence of appropriate documentation around the sedation event

• Cost

• Patient care as defined by trialists: improved success rates for sedation; reduction in waiting times; reduction in inter-hospital transfers; reduction in hospital admissions; and reduction in general anesthetic rates

• Adverse events as defined by trialists: incidence of hypoxia; incidence of hypotension: unscheduled admission to hospital: oversedation leading to prolonged ED stay; failed sedation with inability to complete procedure; and mortality.

### Search methods for identification of studies

We will identify published, unpublished and ongoing studies by searching the following databases:

• The Cochrane Central Register of Controlled Trials (CENTRAL) (1999 (established) to present)

• MEDLINE (OvidSP) (1966 to present)

• Embase (OvidSP) (1980 to present)

• CINAHL (EBSCOhost) (1982 to present)

• Index to Theses

• Web of Knowledge

• Turning Research into Practice (TRIP) database

• ClinicalTrials.gov

• World Health Organization (WHO) International Clinical Trials Registry Platform (ICTRP)

• Australian New Zealand Clinical Trials Registry (ANZCTR)

• Google Scholar.

The search strategy (Appendix 1) will be designed by applying the guidance of the *Cochrane Handbook for Systematic Reviews of Interventions*, Chapter 6.4 [28].

#### Searching other resources

We will also search the reference lists of review articles, relevant trials, textbooks and abstracts of scientific meetings to identify other relevant studies. We will review titles and abstracts to identify all potential trials. We will obtain full text versions of identified articles. Additional efforts will be made to identify other potential studies relevant to this topic from the following data sources:

• International meetings

• Grey literature (theses, internal reports, non-peer reviewed journals) using the OpenGrey database

• References (cited by primary sources)

• Other unpublished sources

• Deep Web search.

We will not impose a language restriction.

### Data collection and analysis

#### Selection of studies

Initial screening of titles and abstracts will be performed by two independent reviewers (SMC and ROS). Disagreement on eligibility will be resolved by discussion/referral to a third party (AW). Full copies of all studies that meet the inclusion criteria will be obtained for further assessment. Second screening of full text journals will be performed by two independent reviewers (MBarrett and AM) applying all inclusion and exclusion criteria outlined in the review protocol.

#### Data extraction and management

Using a data extraction form (DEF) two independent reviewers (MBrenner and CB) will review identified studies for data points identified on the DEF (Additional file 1). Any differences of opinion will be reconciled by mutual agreement. Data will be entered into a database (Review Manager (RevMan), Version 5.2 [29]) by two independent reviewers (SR and AW) for statistical analysis.

### Assessment of risk of bias in included studies

SMC and PL will undertake the risk of bias of the included studies independently, taking the following into consideration, as guided by the *Cochrane Handbook for Systematic Reviews of Interventions*[28]:

• Randomization process

• Performance bias

• Detection bias

• Attrition bias

• Incomplete outcome data

• Selective outcome reporting.

The Cochrane risk of bias tool involves describing each of these domains as reported in the trial and then assigning a judgment about the adequacy of each entry (low risk of bias, high risk of bias and unclear (or unknown) risk of bias) [28]. There are six domains to be examined when using this tool, as follows:

1. Sequence generation;

2. Allocation concealment;

3. Blinding of participants, personnel and outcome;

4. Incomplete outcome data;

5. Selective outcome reporting; and

6. Other sources of bias.

We are aware that potential biases are likely to be greater in non-randomized studies (NRS); the rationale for inclusion of NRS in this review is that we are seeking evidence of effects of interventions that are difficult to randomize.

#### Management of skewed data

The strategies we will use with skewed data depend on the way the original trialists analyze and report results. The options we might encounter include:

• The trialists ignore (or do not notice) the skewness, and simply report means, standard deviations and sample sizes. In this situation, we will directly enter these numbers into RevMan. However, we will be mindful that there is a possibility that this ‘improperly’ analyzed data may be misleading, which may lead to questionable validity of findings.

• The trialists log-transform the data for analysis and report geometric means. When a positively skewed distribution is log-transformed the skewness will be reduced. This is a recommended method of analysis for skewed data. The data we will analyze in RevMan should also be on the log scale: the mean of the logged data will be the log of the geometric data. The standard deviation will be obtained from the confidence interval (CI) for the geometric mean (*Cochrane Handbook for Systematic Reviews of Interventions*, Section 9.4.5.3 [28]).

• The trialists use non-parametric tests (for example Mann–Whitney) and describe averages using medians. The use of non-parametric tests is an alternative for analyzing skewed data in trials. However, we cannot obtain means and standard deviations, and results of such analyses cannot be directly used in a meta-analysis.

• In order to ensure that no data is lost from the review we will report the result of all studies, regardless of the method of analysis in a table. This means results will be considered when drawing conclusions, even if they cannot be formally pooled.

#### Measures of treatment effect

When the measure of the outcome is sufficiently consistent across trials, we will use odds ratios (ORs) for dichotomous data and mean difference (MD) for continuous data with corresponding 95% CIs.

#### Unit/scale of analysis issues

The unit of analysis is based on the individual patient (the unit to be randomized for interventions to be compared). For any included trials using a crossover design, we will use only pre-crossover data. Analysis of NRS will follow a similar methodology to the analysis of RCTs with particular attention focused on allocation methods, as this is likely to give rise to an increased risk of selection bias. Limiting the inclusion criteria of NRS by study design or methodological quality could potentially reduce bias (as NRS design influences susceptibility to bias). Raw results from NRS are not as clear as RCTs as the raw data is unadjusted and susceptible to confounding. Therefore, an adjusted comparison will be reported using a regression model. Any adjusted effect estimates and standard errors/CIs will be documented. The adjusted effect estimates and their precision can be displayed in forest plots. This is suitable for studies that are not sufficiently homogeneous to combine in a meta-analysis if appropriate data across studies can be pooled. Cluster randomized trials have in the past failed to report appropriate analyses. They are commonly analyzed as if the randomization was performed on the individuals rather than the clusters. If this is the situation, approximately correct analyses may be performed if the following information can be extracted:

1. The number of clusters (or groups) randomized to each intervention group; or the average (mean) size of each cluster;

2. The outcome data ignoring the cluster design for the total number of individuals (for example, number or proportion of individuals with events, or means and standard deviations); and

3. An estimate of the intracluster (or intraclass) correlation coefficient (ICC).

The ICC is an estimate of the relative variability within and between clusters [30]. It describes the ‘similarity’ of individuals within the same cluster. In fact, this is seldom available in published reports. A common approach is to use external estimates obtained from similar studies, and several resources are available that provide examples of ICCs [31,32]. ICCs may appear small compared with other types of correlations: values lower than 0.05 are typical. However, even small values can have a substantial impact on CI widths (and hence weights in a meta-analysis), particularly if cluster sizes are large. Empirical research has observed that larger cluster sizes are associated with smaller ICCs [31].

An approximately correct analysis proceeds as follows. The idea is to reduce the size of each trial to its ‘effective sample size’ [33]. The effective sample size of a single intervention group in a cluster randomized trial is its original sample size divided by a quantity called the ‘design effect’. The design effect is 1 + (M – 1) × ICC, where M is the average cluster size and ICC is the intracluster correlation coefficient. A common design effect is usually assumed across intervention groups. The analysis of cluster randomized trials will be assessed at the outcomes at the level of group, and the unit of analysis will be the same as the unit of randomization. Measurements can be dichotomous outcomes, such as success/failure or a continuous outcome utilizing a percentage of individuals in the group who benefited. For dichotomous data both the number of participants and the number experiencing the event should be divided by the same design effect. Since the resulting data must be rounded to whole numbers for entry into RevMan this approach may be unsuitable for small trials. For continuous data only the sample size need be reduced; means and standard deviations should remain unchanged. This will provide one outcome measurement from each randomized unit so analysis can proceed as if the groups were individuals.

#### Dealing with missing data

Where data seems to be missing from the study, this will, if possible, be obtained by correspondence with the study authors.

### Assessment of heterogeneity

We will evaluate clinical heterogeneity (GOC and AW), and will apply statistical and qualitative methods. To assess statistical heterogeneity we will apply the chi-squared test, as a low *P* value is evidence of the heterogeneity of intervention effects. In addition to statistical assessments we will review studies examining variability in study participants, interventions and outcomes as this would be suggestive of heterogeneity. In the absence of clinical heterogeneity we will use the I^2^ statistic to describe the percentage of total variation across studies that is due to the heterogeneity rather than chance. An I^2^ value greater than 50% will be considered significant heterogeneity. We will also use visual inspection of the graphical representation of study results with their 95% CIs to assess heterogeneity.

### Assessment of reporting biases

Detecting publication bias is difficult and avoidance is a better strategy [34]. We will avoid publication bias by comprehensive literature searching and use of study registries [34]. The authors will be obtaining and including data from unpublished work and no language restriction will be imposed reducing the risk of reporting bias. We will use a graphical display (funnel plot if greater than ten studies are included) of the size of treatment effect against the precision of the trial to investigate publication bias by looking for signs of asymmetry. Publication bias is associated with asymmetry [35]. If there is asymmetry, we will look for reasons other than publication bias.

### Data synthesis

The results will concentrate on the objectives and comparisons specified in the protocol of the review. Post-hoc analysis will be identified as such. We will analyze results using both fixed-effect and random-effect meta-analysis, because for each model there are situations where the result is counterintuitive. We will use the fixed-effect model meta-analysis except where statistical heterogeneity is identified, in which case we will use the random-effects model [36]. We will consider the appropriateness of meta-analysis in the presence of significant clinical or statistical heterogeneity. We will perform the meta-analyses using RevMan software (Version 5.2).

### Data analysis

On the basis of quality appraisal, SMC and ROS will perform a data analysis. First, providing a table with a simple descriptive evaluation of each study, including the following:

• Population under study

• Interventions

• Methods

• Biases

• Outcomes

From these tables it can be determined if the results from the studies can be pooled and subjected to a meta-analysis.

### Subgroup analysis

A subgroup analysis will be performed on:

• Different types of structured sedation program: theoretical-based learning only; and theoretical and practice-based learning

• ‘High risk’ populations: children (defined as <18 years); children (<2 years); children with developmental delay/autism spectrum disorders (ASD); and older adults (defined as >65 years).

### Time points

Data will be analyzed for immediate impact of a program and sustainability of change in line with previously identified outcomes. The time frame for immediate impact will be data reported 3 months post-introduction of a program and sustainability will be measured at time points greater than or equal to 12 months. We will consider combining data extracted within these parameters.

### Interpretation of results

Results will be presented in tabular form. At this point the strengths and weaknesses of each study will be discussed, and recommendations made for future studies by identifying knowledge-deficient areas within the subject area.

## Discussion

This review will cohere evidence on the effectiveness of structured PSPs on sedation events and patient outcomes within the hospital and other acute care settings. In addition, it will examine key components identified within a PSP associated with patient safety and improved patient outcomes.

## Appendix 1 Search strategy

1. Procedural sedation

2. Twilight anesthesia

3. Conscious sedation

4. Moderate sedation

5. Minimal sedation

6. Dissociation

7. Anesthesia/anaesthesia

8. #1 OR #2 OR #3 OR #4 OR #5 OR #6 NOT #7

9. #8 NOT animals

10. #9 AND (program development OR education program OR program evaluation)

## Abbreviations

AAP: American Academy of Pediatrics; ANZCTR: Australian New Zealand Clinical Trials Registry; ASD: Autism spectrum disorders; CBA: Controlled before and after; CI: Confidence interval; CPG: Clinical practice guideline; C-RCT: Cluster randomized controlled trial; DEF: Data extraction form; ED: Emergency department; ICC: Intracluster correlation coefficient; ICTRP: International Clinical Trials Registry Platform; ITS: Interrupted time series; MD: Mean difference; NRCT: Non-randomized control trial; NRS: Non-randomized studies: OR, odds ratio; PREDICT: Paediatric Research in Emergency Departments International Collaborative; PSP: Procedural sedation program; RCT: Randomized controlled trial; RevMan: Review Manager; TRIP: Turning Research into Practice; WHO: World Health Organization.

## Competing interests

The authors have no competing interests.

## Authors’ contributions

SMC and ROS conceptualized the study, designed the study protocol, and drafted the protocol. SMC and ROS will participate in the first screening of identified studies, assessment of risk of bias, and final data analysis. SMC will draft the final review, while ROS will edit the finalized manuscript. MBarrett contributed to the design of the study protocol and the search strategy, and will review full text articles and edit the finalized manuscript. CB contributed to design of the study protocol, and will participate in data extraction and editing of the finalized manuscript. AM will review the full text articles and edit the finalized manuscript. PL will perform the risk of bias assessment, contributed to the design of the study protocol and will edit the finalized manuscript. MBrenner will perform data extraction, has contributed to the design of study protocol and will edit the finalized manuscript. AW participated in the design of the study, and will contribute to the statistical analysis and editing of the finalized manuscript. SR will participate in data entry, assisted in design of the study protocol and will edit the finalized manuscript. GOC will perform the statistical analysis. All authors read and approved the final manuscript.

## Supplementary Material

Additional file 1 Data Extraction Form. Click here for file
