# Supplementary material for: Structured sedation programs in the emergency department, hospital and other acute settings: protocol for systematic review of effects and events
Source: Syst Rev. 2013 Oct 1;2:89. doi: 10.1186/2046-4053-2-89 (PMC3850685; doi:10.1186/2046-4053-2-89)
Supplement: Additional file 1 — Data Extraction Form. [file 2046-4053-2-89-S1.doc]

Appendix 2 ***Data Extraction Form***

| **Title of Study** |  |
| --- | --- |
| **Source (Journal; Year; Volume; Pages)** |  |
| **Authors** |  |
| **Language of Publication** |  |
| **Type of Report (Full paper/abstracts/unpublished)** |  |

| **Characteristics of trial** | |
| --- | --- |
| Country where trial was conducted |  |
| Funders of trial |  |
| Date trial was conducted |  |
| Was the trial multi-centre? If so, how many centres? |  |

| **Characteristics of the Participants** | |
| --- | --- |
| Inclusion Criteria |  |
| Exclusion Criteria |  |
| Total number of randomised participants |  |
| total available for analysis |  |
| information on the age of the participants |  |
| information on the sex of the participants |  |
| information on the ethnicity of the participants |  |

| **Intervention Group** | |
| --- | --- |
| Intervention |  |
| Number of participants |  |
| Where additional interventions given to this group |  |

| **Control Group** | |
| --- | --- |
| Control |  |
| Number of participants |  |
| Where additional interventions given to this group |  |

| **Outcome Measures** | **No Sedation Programme** | **Sedation Programme** |
| --- | --- | --- |
| Respiratory or airway compromise requiring ventilatory support |  |  |
| Patient satisfaction rates as defined by study authors |  |  |
| Practitioner Competence   - Increased Knowledge - Increased Skills - Documented attitude/ behaviour changes - Increased Number of successful sedations - Reduction in overall adverse events - Self- Reported confidence/attitude/behaviour Change |  |  |
| Evidence of appropriate documentation around the sedation event |  |  |
| Cost |  |  |
| Adverse Event as defined by triallist   - Incidence of Hypoxia - Incidence of Hypotension - Unscheduled admission to hospital - Oversedation leading to prolonged ED stay - Failed sedation with inability to complete procedure - Mortality |  |  |
